# 4-Amino-3-(*p*-tolyl­oxymeth­yl)-1*H*-1,2,4-triazole-5(4*H*)-thione

**DOI:** 10.1107/S1600536809027664

**Published:** 2009-07-18

**Authors:** Hoong-Kun Fun, Jia Hao Goh, A. M. Vijesh, Mahesh Padaki, Arun M. Isloor

**Affiliations:** a X-ray Crystallography Unit, School of Physics, Universiti Sains Malaysia, 11800 USM, Penang, Malaysia; b SeQuent Scientific Limited, No. 120 A&B, Industrial Area, Baikampady, New Mangalore, Karnataka 575 011, India; c Department of Chemistry, National Institute of Technology-Karnataka, Surathkal, Mangalore 575 025, India

## Abstract

In the title triazole compound, C_10_H_12_N_4_OS, the triazole ring is essentially planar [maximum deviation = 0.009 (1) Å] and forms a dihedral angle of 5.78 (4)° with the benzene ring. In the crystal structure, mol­ecules are linked into dimers by centrosymmetric N—H⋯S inter­actions. These dimers are linked into two-mol­ecule-wide tapes by N—H⋯N and S⋯S [3.2634 (3) Å] inter­actions. In addition, they are further inter­connected by weak N—H⋯S inter­actions into sheets parallel to the *ab* plane. The crystal structure is further stabilized by weak inter­molecular C—H⋯π inter­actions.

## Related literature

For general background and applications of triazole derivatives, see: Amir *et al.* (2008 ▶); Kuş *et al.* (2008 ▶); Krzysztof *et al.* (2008 ▶); Padmavathi *et al.* (2008 ▶). For the preparation, see: Conti (1964 ▶). For related structures, see: Fun *et al.* (2008 ▶, 2009 ▶). For the stability of the temperature controller used for the data collection, see: Cosier & Glazer (1986 ▶).
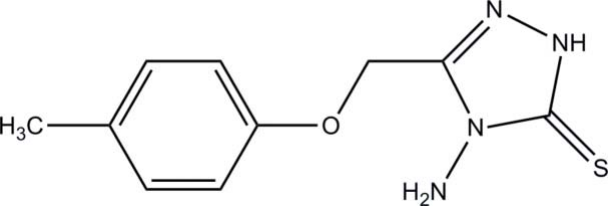


## Experimental

### 

#### Crystal data

C_10_H_12_N_4_OS
*M* *_r_* = 236.30
Triclinic, 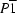

*a* = 5.9977 (1) Å
*b* = 6.4002 (1) Å
*c* = 15.5506 (2) Å
α = 89.352 (1)°
β = 83.157 (1)°
γ = 65.562 (1)°
*V* = 539.11 (1) Å^3^
*Z* = 2
Mo *K*α radiation
μ = 0.28 mm^−1^
*T* = 100 K
0.47 × 0.30 × 0.09 mm

#### Data collection

Bruker SMART APEXII CCD area-detector diffractometer
Absorption correction: multi-scan (**SADABS**; Bruker, 2005 ▶) *T*_min_ = 0.878, *T*_max_ = 0.975
19910 measured reflections
4712 independent reflections
4228 reflections with *I* > 2σ(*I*)
*R*_int_ = 0.022

#### Refinement

*R*[*F* ^2^ > 2σ(*F* ^2^)] = 0.032
*wR*(*F* ^2^) = 0.095
*S* = 1.05
4712 reflections
193 parameters
H atoms treated by a mixture of independent and constrained refinement
Δρ_max_ = 0.59 e Å^−3^
Δρ_min_ = −0.33 e Å^−3^

### 

Data collection: *APEX2* (Bruker, 2005 ▶); cell refinement: *SAINT* (Bruker, 2005 ▶); data reduction: *SAINT*; program(s) used to solve structure: *SHELXTL* (Sheldrick, 2008 ▶); program(s) used to refine structure: *SHELXTL*; molecular graphics: *SHELXTL*; software used to prepare material for publication: *SHELXTL* and *PLATON* (Spek, 2009 ▶).

## Supplementary Material

Crystal structure: contains datablocks global, I. DOI: 10.1107/S1600536809027664/tk2497sup1.cif

Structure factors: contains datablocks I. DOI: 10.1107/S1600536809027664/tk2497Isup2.hkl

Additional supplementary materials:  crystallographic information; 3D view; checkCIF report

## Figures and Tables

**Table 1:** Hydrogen-bond geometry (Å, °)

*D*—H⋯*A*	*D*—H	H⋯*A*	*D*⋯*A*	*D*—H⋯*A*
N2—H1*N*2⋯S1^i^	0.930 (13)	2.412 (13)	3.3364 (7)	172.5 (11)
N4—H1*N*4⋯N1^ii^	0.930 (17)	2.428 (17)	3.2100 (9)	141.6 (12)
N4—H2*N*4⋯S1^iii^	0.894 (15)	2.937 (16)	3.5456 (7)	126.8 (12)
C10—H10*C*⋯*Cg*2^iv^	0.968 (18)	2.697 (19)	3.6250 (9)	160.8 (14)

Symmetry codes: (i) 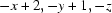; (ii) 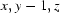; (iii) 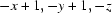; (iv) 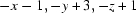. *Cg*2 is the centroid of the C4–C9 ring.

**Fig. 1.**
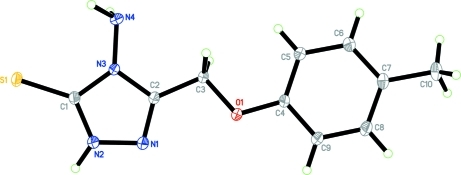
The molecular structure of (I), showing 50% probability displacement ellipsoids and the atom-numbering scheme.

**Fig. 2.**
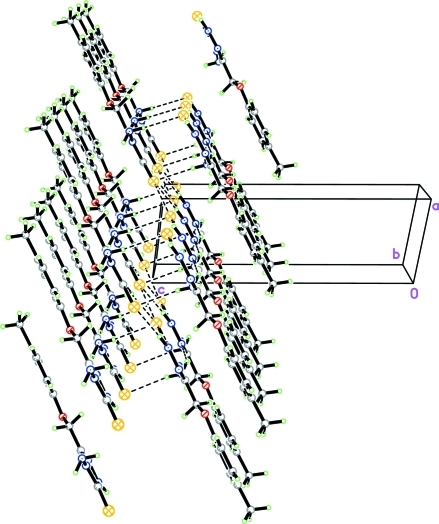
Two-molecular-wide tapes connected by N—H···S, N—H···N and S···S interactions. The tapes form sheets parallel to the ab plane via weaker N—H···S interactions. Intermolecular interactions are shown as dashed bonds.

## supplementary crystallographic information

### Comment

1,2,4-Triazole and its derivatives were reported to exhibit various pharmacological activities such as anti-microbial, analgesic, anti-inflammatory, anti-cancer and anti-oxidant properties (Amir *et al.*, 2008; Kuş *et al.*, 2008; Krzysztof *et al.*, 2008; Padmavathi *et al.*, 2008). Some of the present day drugs such as Ribavirin (anti-viral agent), Rizatriptan (anti-migraine agent), Alprazolam (anxiolytic agent), Fluconazole and Itraconazole (anti-fungal agents) are the best examples for potent molecules possessing the triazole nucleus. The amino and mercapto groups of thio-substituted 1,2,4-triazole serve as readily accessible nucleophilic centers for the preparation of N-bridged heterocycles. In view of their biological importance, we have synthesized the title compound (I) to study its crystal structure.

In (I), Fig. 1, the 1,2,4-triazole ring (C1/C2/N1-N3) is essentially planar, with a maximum deviation of 0.009 (1) Å for atom C1. The 1,2,4-triazole ring makes dihedral angle of 5.78 (4)° with the C4-C9 benzene ring. The bond lengths and angles in the molecule are comparable to those found in closely related structures (Fun *et al.*, 2008, 2009).

In the crystal packing (Fig. 2), centrosymmetrically related molecules are linked into dimers by N2—H1N2···S1 interactions (Table 1). These dimers are linked into two-molecule-wide tapes by N4—H1N4···N1 (Table 1) and by short S1···S1 contacts of 3.2634 (3) Å; symmetry code: 2-x, -y, -z. In addition, these tapes are interconnected into sheets parallel to the *ab* plane by weak N4—H2N4···S1 interactions (Table 1). The crystal structure is further stabilized by weak C···H···π interactions (Table 1).

### Experimental

P-Cresoloxyacetyl hydrazine (18.0 g, 0.10 mol) was added slowly to a solution of potassium hydroxide (8.4 g, 0.15 mol) in ethanol (150 ml). The resulting mixture was stirred well until a clear solution was obtained. Carbon disulphide (11.4 g, 0.15 mol) was added drop-wise and the contents were stirred vigorously. Further stirring was continued for 24 h. The resulting mixture was diluted with ether (100 ml) and the precipitate formed was collected by filtration, washed with dry ether and dried at 65 °C under vacuum. It was used for the next step without any purification.

A mixture of the above synthesized potassium dithiocarbazinate (29.4 g, 0.10 mol), hydrazine hydrate (99 %, 0.20 mol) and water (2 ml) was heated gently to boil for 30 minutes. Heating was continued until the evacuation of hydrogen sulphide ceased. The reaction mixture was cooled to room temperature, diluted with water (100 ml) and acidified with HCl. The solid mass that separated was collected by filtration, washed with water and dried. Recrystallization was achieved from ethanol (Conti, 1964). The yield was 14.63 g, 62 %. *M.p.* 461-463 K.

### Refinement

All the H atoms were located from difference Fourier map [range of C-H = 0.894 (15) - 1.011 (12) Å] and allowed to refine freely.

### Crystal data

**Table:**

C_10_H_12_N_4_OS	*Z* = 2
*M**_r_* = 236.30	*F*(000) = 248
Triclinic, *P*1	*D*_x_ = 1.456 Mg m^−^^3^
Hall symbol: -P 1	Mo *K*α radiation, λ = 0.71073 Å
*a* = 5.9977 (1) Å	Cell parameters from 9925 reflections
*b* = 6.4002 (1) Å	θ = 2.6–35.2°
*c* = 15.5506 (2) Å	µ = 0.28 mm^−^^1^
α = 89.352 (1)°	*T* = 100 K
β = 83.157 (1)°	Plate, colourless
γ = 65.562 (1)°	0.47 × 0.30 × 0.09 mm
*V* = 539.11 (1) Å^3^	

### Data collection

**Table:**

Bruker SMART APEXII CCD area-detector diffractometer	4712 independent reflections
Radiation source: fine-focus sealed tube	4228 reflections with *I* > 2σ(*I*)
graphite	*R*_int_ = 0.022
φ and ω scans	θ_max_ = 35.0°, θ_min_ = 2.6°
Absorption correction: multi-scan (*SADABS*; Bruker, 2005)	*h* = −9→9
*T*_min_ = 0.878, *T*_max_ = 0.975	*k* = −10→10
19910 measured reflections	*l* = −25→24

### Refinement

**Table:**

Refinement on *F*^2^	Primary atom site location: structure-invariant direct methods
Least-squares matrix: full	Secondary atom site location: difference Fourier map
*R*[*F*^2^ > 2σ(*F*^2^)] = 0.032	Hydrogen site location: inferred from neighbouring sites
*wR*(*F*^2^) = 0.095	H atoms treated by a mixture of independent and constrained refinement
*S* = 1.05	*w* = 1/[σ^2^(*F*_o_^2^) + (0.0648*P*)^2^ + 0.0513*P*] where *P* = (*F*_o_^2^ + 2*F*_c_^2^)/3
4712 reflections	(Δ/σ)_max_ = 0.001
193 parameters	Δρ_max_ = 0.59 e Å^−^^3^
0 restraints	Δρ_min_ = −0.33 e Å^−^^3^

### Special details

**Table:**

Experimental. The crystal was placed in the cold stream of an Oxford Cyrosystems Cobra open-flow nitrogen cryostat (Cosier & Glazer, 1986) operating at 100.0 (1)K.
Geometry. All esds (except the esd in the dihedral angle between two l.s. planes) are estimated using the full covariance matrix. The cell esds are taken into account individually in the estimation of esds in distances, angles and torsion angles; correlations between esds in cell parameters are only used when they are defined by crystal symmetry. An approximate (isotropic) treatment of cell esds is used for estimating esds involving l.s. planes.
Refinement. Refinement of F^2^ against ALL reflections. The weighted R-factor wR and goodness of fit S are based on F^2^, conventional R-factors R are based on F, with F set to zero for negative F^2^. The threshold expression of F^2^ > 2sigma(F^2^) is used only for calculating R-factors(gt) etc. and is not relevant to the choice of reflections for refinement. R-factors based on F^2^ are statistically about twice as large as those based on F, and R- factors based on ALL data will be even larger.

### Fractional atomic coordinates and isotropic or equivalent isotropic displacement parameters (Å^2^)

**Table:**

	*x*	*y*	*z*	*U*_iso_*/*U*_eq_	
S1	0.84328 (3)	0.26155 (3)	0.040453 (11)	0.01525 (6)	
O1	0.06661 (10)	1.12912 (9)	0.24927 (4)	0.01568 (10)	
N1	0.49686 (12)	0.89098 (11)	0.14036 (4)	0.01591 (11)	
N2	0.69184 (11)	0.71323 (11)	0.09371 (4)	0.01581 (11)	
H1N2	0.820 (2)	0.732 (2)	0.0596 (9)	0.024 (3)*	
N3	0.43624 (11)	0.57330 (10)	0.13942 (4)	0.01199 (10)	
N4	0.30133 (12)	0.43979 (11)	0.15295 (4)	0.01506 (11)	
H1N4	0.405 (2)	0.293 (3)	0.1674 (9)	0.023 (3)*	
H2N4	0.258 (3)	0.419 (3)	0.1018 (10)	0.035 (4)*	
C1	0.66067 (12)	0.51754 (12)	0.09031 (4)	0.01305 (11)	
C2	0.34451 (12)	0.79838 (12)	0.16710 (4)	0.01274 (11)	
C3	0.09665 (12)	0.91116 (11)	0.21854 (5)	0.01325 (11)	
H3A	−0.034 (2)	0.927 (2)	0.1804 (8)	0.018 (3)*	
H3B	0.086 (2)	0.808 (2)	0.2649 (8)	0.016 (3)*	
C4	−0.16049 (12)	1.26194 (12)	0.29390 (4)	0.01307 (12)	
C5	−0.34570 (13)	1.18698 (13)	0.31707 (5)	0.01573 (12)	
H5A	−0.333 (2)	1.039 (2)	0.2991 (9)	0.026 (3)*	
C6	−0.56524 (13)	1.33505 (13)	0.36639 (5)	0.01718 (13)	
H6A	−0.682 (3)	1.275 (2)	0.3802 (9)	0.027 (3)*	
C7	−0.60421 (13)	1.55609 (12)	0.39238 (5)	0.01587 (13)	
C8	−0.41667 (15)	1.62850 (13)	0.36627 (5)	0.01808 (13)	
H8A	−0.443 (3)	1.785 (3)	0.3837 (9)	0.028 (3)*	
C9	−0.19792 (14)	1.48515 (12)	0.31738 (5)	0.01693 (13)	
H9A	−0.070 (3)	1.533 (3)	0.2975 (10)	0.031 (4)*	
C10	−0.83720 (14)	1.71372 (15)	0.44753 (5)	0.02093 (15)	
H10A	−0.902 (3)	1.871 (3)	0.4283 (11)	0.042 (4)*	
H10B	−0.971 (3)	1.672 (3)	0.4484 (11)	0.046 (4)*	
H10C	−0.807 (3)	1.720 (3)	0.5070 (12)	0.049 (5)*	

### Atomic displacement parameters (Å^2^)

**Table:**

	*U*^11^	*U*^22^	*U*^33^	*U*^12^	*U*^13^	*U*^23^
S1	0.01469 (9)	0.01076 (8)	0.01619 (9)	−0.00247 (6)	0.00295 (6)	−0.00124 (6)
O1	0.0137 (2)	0.0113 (2)	0.0208 (2)	−0.00540 (17)	0.00364 (18)	−0.00495 (18)
N1	0.0146 (2)	0.0125 (2)	0.0193 (3)	−0.0056 (2)	0.0033 (2)	−0.0034 (2)
N2	0.0138 (2)	0.0134 (2)	0.0194 (3)	−0.0062 (2)	0.0036 (2)	−0.0032 (2)
N3	0.0118 (2)	0.0090 (2)	0.0141 (2)	−0.00392 (18)	0.00088 (18)	−0.00093 (18)
N4	0.0165 (3)	0.0110 (2)	0.0186 (3)	−0.0073 (2)	0.0007 (2)	0.0000 (2)
C1	0.0118 (2)	0.0120 (3)	0.0136 (3)	−0.0037 (2)	0.0004 (2)	−0.0002 (2)
C2	0.0130 (2)	0.0099 (2)	0.0139 (3)	−0.0038 (2)	0.0002 (2)	−0.0013 (2)
C3	0.0126 (3)	0.0095 (2)	0.0156 (3)	−0.0035 (2)	0.0018 (2)	−0.0025 (2)
C4	0.0125 (3)	0.0106 (3)	0.0143 (3)	−0.0035 (2)	0.0008 (2)	−0.0015 (2)
C5	0.0140 (3)	0.0128 (3)	0.0195 (3)	−0.0055 (2)	0.0011 (2)	−0.0024 (2)
C6	0.0134 (3)	0.0163 (3)	0.0199 (3)	−0.0051 (2)	0.0015 (2)	−0.0026 (2)
C7	0.0141 (3)	0.0143 (3)	0.0147 (3)	−0.0017 (2)	−0.0003 (2)	−0.0011 (2)
C8	0.0192 (3)	0.0113 (3)	0.0204 (3)	−0.0041 (2)	0.0018 (2)	−0.0028 (2)
C9	0.0179 (3)	0.0117 (3)	0.0201 (3)	−0.0064 (2)	0.0028 (2)	−0.0026 (2)
C10	0.0164 (3)	0.0199 (3)	0.0193 (3)	−0.0011 (3)	0.0014 (2)	−0.0041 (3)

### Geometric parameters (Å, °)

**Table:**

S1—C1	1.6812 (7)	C4—C5	1.3913 (10)
O1—C4	1.3746 (8)	C4—C9	1.3970 (10)
O1—C3	1.4119 (9)	C5—C6	1.4012 (10)
N1—C2	1.3080 (9)	C5—H5A	0.962 (14)
N1—N2	1.3798 (9)	C6—C7	1.3915 (10)
N2—C1	1.3432 (9)	C6—H6A	0.930 (14)
N2—H1N2	0.932 (13)	C7—C8	1.4011 (11)
N3—C2	1.3657 (9)	C7—C10	1.5078 (10)
N3—C1	1.3734 (9)	C8—C9	1.3868 (10)
N3—N4	1.3988 (8)	C8—H8A	0.984 (14)
N4—H1N4	0.928 (14)	C9—H9A	0.959 (14)
N4—H2N4	0.894 (15)	C10—H10A	0.975 (17)
C2—C3	1.4875 (9)	C10—H10B	0.944 (17)
C3—H3A	1.011 (12)	C10—H10C	0.968 (18)
C3—H3B	0.986 (12)		
			
C4—O1—C3	115.53 (5)	O1—C4—C9	115.49 (6)
C2—N1—N2	103.33 (6)	C5—C4—C9	119.96 (6)
C1—N2—N1	113.75 (6)	C4—C5—C6	119.23 (7)
C1—N2—H1N2	121.9 (9)	C4—C5—H5A	122.9 (8)
N1—N2—H1N2	123.5 (9)	C6—C5—H5A	117.8 (8)
C2—N3—C1	108.49 (6)	C7—C6—C5	121.82 (7)
C2—N3—N4	122.95 (6)	C7—C6—H6A	122.7 (9)
C1—N3—N4	128.18 (6)	C5—C6—H6A	115.4 (9)
N3—N4—H1N4	109.3 (8)	C6—C7—C8	117.60 (7)
N3—N4—H2N4	107.2 (10)	C6—C7—C10	122.01 (7)
H1N4—N4—H2N4	104.5 (13)	C8—C7—C10	120.39 (7)
N2—C1—N3	102.99 (6)	C9—C8—C7	121.62 (7)
N2—C1—S1	130.61 (6)	C9—C8—H8A	120.1 (8)
N3—C1—S1	126.40 (5)	C7—C8—H8A	118.3 (8)
N1—C2—N3	111.41 (6)	C8—C9—C4	119.73 (7)
N1—C2—C3	127.78 (6)	C8—C9—H9A	122.7 (9)
N3—C2—C3	120.78 (6)	C4—C9—H9A	117.5 (9)
O1—C3—C2	108.32 (6)	C7—C10—H10A	112.9 (10)
O1—C3—H3A	110.4 (7)	C7—C10—H10B	114.7 (11)
C2—C3—H3A	109.3 (7)	H10A—C10—H10B	104.2 (14)
O1—C3—H3B	113.9 (7)	C7—C10—H10C	110.4 (11)
C2—C3—H3B	107.8 (7)	H10A—C10—H10C	107.1 (14)
H3A—C3—H3B	107.0 (10)	H10B—C10—H10C	107.1 (15)
O1—C4—C5	124.54 (6)		
			
C2—N1—N2—C1	0.94 (8)	N1—C2—C3—O1	−11.09 (10)
N1—N2—C1—N3	−1.56 (8)	N3—C2—C3—O1	171.04 (6)
N1—N2—C1—S1	178.86 (6)	C3—O1—C4—C5	6.66 (10)
C2—N3—C1—N2	1.55 (8)	C3—O1—C4—C9	−174.38 (6)
N4—N3—C1—N2	174.57 (7)	O1—C4—C5—C6	176.82 (7)
C2—N3—C1—S1	−178.85 (5)	C9—C4—C5—C6	−2.10 (11)
N4—N3—C1—S1	−5.83 (11)	C4—C5—C6—C7	0.49 (12)
N2—N1—C2—N3	0.13 (8)	C5—C6—C7—C8	1.01 (11)
N2—N1—C2—C3	−177.90 (7)	C5—C6—C7—C10	−178.28 (7)
C1—N3—C2—N1	−1.10 (8)	C6—C7—C8—C9	−0.94 (12)
N4—N3—C2—N1	−174.56 (6)	C10—C7—C8—C9	178.36 (7)
C1—N3—C2—C3	177.09 (6)	C7—C8—C9—C4	−0.64 (12)
N4—N3—C2—C3	3.62 (10)	O1—C4—C9—C8	−176.84 (7)
C4—O1—C3—C2	176.18 (6)	C5—C4—C9—C8	2.18 (12)

### Hydrogen-bond geometry (Å, °)

**Table:**

*D*—H···*A*	*D*—H	H···*A*	*D*···*A*	*D*—H···*A*
N2—H1N2···S1^i^	0.930 (13)	2.412 (13)	3.3364 (7)	172.5 (11)
N4—H1N4···N1^ii^	0.930 (17)	2.428 (17)	3.2100 (9)	141.6 (12)
N4—H2N4···S1^iii^	0.894 (15)	2.937 (16)	3.5456 (7)	126.8 (12)
C10—H10C···Cg2^iv^	0.968 (18)	2.697 (19)	3.6250 (9)	160.8 (14)

Symmetry codes: (i) −*x*+2, −*y*+1, −*z*; (ii) *x*, *y*−1, *z*; (iii) −*x*+1, −*y*+1, −*z*; (iv) −*x*−1, −*y*+3, −*z*+1.
